# The value of maintaining social connections for mental health in older people

**DOI:** 10.1016/S2468-2667(19)30253-1

**Published:** 2020-01

**Authors:** Michelle G Newman, Nur Hani Zainal

**Affiliations:** Department of Psychology, The Pennsylvania State University, University Park, PA 16802-3103, USA

By 2050, it is estimated that about a fifth of the general population will be aged 65 years and older.^1^ Social isolation and loneliness among young (18–40 years), middle-aged (41–64), and older adults (65 years and older) is thus a serious public health concern of our time because of its strong connection with cardiovascular, autoimmune, neurocognitive, and mental health problems.^2^ The scientific literature has documented the bidirectional and complex relationship between psychological issues and social disconnectedness in the past 40 years.^3^ Despite extensive work done to date on this topic, previous research has had several shortcomings. Limitations include preponderance of cross-sectional data that precludes causal inferences, use of single measure or single-item assessments of loneliness, absence of testing bidirectionality, and small sample sizes.

In *The Lancet Public Health*, Ziggi Ivan Santini and colleagues^4^ build on previous work by examining the bidirectional relations between depression or anxiety severity and social disconnectedness between 2005 and 2016, and the degree to which perceived isolation mediated those relationships. The authors tested these hypotheses in a large sample of 3005 community-dwelling adults aged 57 to 85 years in the National Social Life, Health, and Aging Project using random-intercept cross-lagged panel modelling.^5^ The analyses showed that social disconnectedness independently predicted depression and anxiety symptom severity (and vice versa). Additionally, self-perceived social isolation was found to mediate the link between social disconnectedness and depression and anxiety in both directions. For example, social disconnectedness predicted higher subsequent perceived isolation, which in turn predicted higher depression symptoms and anxiety symptoms (all p<0.0001). The random-intercept cross-lagged panel modelling approach, which adjusts for previous outcomes and between-person variation, permits the inference that these observed relations unfold within (as opposed to between) people, thus bringing us closer toward causal models. Moreover, the authors exemplified the best practices of longitudinal structural equation modelling by testing for measurement equivalence to verify that the latent constructs were assessed along the same scale at various timepoints (an often-neglected step).

These findings can potentially inform public health and social policies. Brief evidence-based preventive interventions could plausibly be developed for older adults and implemented within multiple healthcare venues, religious or cultural organisations, and community centres. Such skills could help older adults form meaningful connections with others. Cognitive skills could help them to critically evaluate the degree to which their social support network fulfills their need for friendships and a sense of belonging. Relatedly, action-based strategies, such as establishing more frequent social contact with significant others or repairing strained relationships, might be important to deliver the best quality care. Randomised controlled trials examining the effects of cognitive behavioural based therapies, delivered online or in-person, have been shown to alleviate depression and anxiety symptoms while simultaneously decreasing loneliness.^7,8^ For instance, internet-delivered cognitive behaviourial therapy has been shown to enhance both the general impression and tangible indicators of social affiliation and support compared with waitlist controls.^7,9^ Similarly, establishing community volunteer outreach could also help in this regard, particularly for adults who are less mobile or more secluded.

These approaches can be implemented in geriatric and other clinical contexts, as well as welfare organisations that provide a range of meaningful and health-promoting social activities to older adults. Second, health-care providers can benefit from being mindful of the potentially scarring effects of untreated depressive and anxiety disorders in middle and late adulthood. Late-life affective disorders can trigger vicious cycles of social withdrawal, unhelpful self-referential thought patterns, and worsened psychiatric symptoms in the long term. Collectively, findings suggest that access to ageing or retirement communities that provide a sense of belonging and security is imperative for delivering high standards of mental health care to older adults.^6^

However, some study limitations deserve mention. It remains unclear if the results would be replicated if diagnostic or clinician rated measures (as opposed to self-report) were used; future replication efforts could thus administer multimodal diagnostic psychiatric instruments. In addition, although the prospective, observational design approximates causality by establishing temporal precedence and co-variation, no strong causal conclusions can be drawn because of the absence of experimental manipulation required for internal validity.

Finally, it is important to keep in mind that most psychological treatments targeting loneliness and related constructs to date were limited by small sample size, non-randomised controlled designs (eg, prepost effectiveness trials), absence of multiple-domain assessments of social disconnectedness, and use of an inactive no-treatment or waitlist comparison group. Accordingly, public health can benefit from future studies using a randomised controlled trial design with a bigger sample size, alongside a multiple-group factorial design to tease apart the treatments’ causal mechanisms. Further, an active control comparison group should be used to rule out regression to the mean, expectancy effects, and other confounders, to analyse factors that contribute to treatment effects on change in outcomes. For example, it might be worth examining if the remedying effect of cognitive behavioural treatments on social disconnectedness might be mediated or moderated by changes in allostatic load, immune functioning, lifestyle, or other putative variables.^10^ Nonetheless, the study by Santini and colleagues is an important first step toward understanding the importance of social support for older adults in helping to prevent depression or anxiety.

## References

[1] Chang AY, Skirbekk VF, Tyrovolas S, Kassebaum NJ, Dieleman JL. Measuring population ageing: an analysis of the Global Burden of Disease Study 2017 Lancet Public Health 2019; 4: e159–67 30851869 10.1016/S2468-2667(19)30019-2 PMC6472541

[2] Gerst-Emerson K, Jayawardhana J. Loneliness as a public health issue: the impact of loneliness on health care utilization among older adults. Am J Public Health 2015; 105: 1013–19. 25790413 10.2105/AJPH.2014.302427 PMC4386514

[3] Klinenberg E Social isolation, loneliness, and living alone: identifying the risks for public health. Am J Public Health 2016; 106: 786–87. 27049414 10.2105/AJPH.2016.303166 PMC4985072

[4] Santini ZI, Jose PE, York Cornwell E, Social disconnectedness, perceived isolation, and symptoms of depression and anxiety among older Americans (NSHAP): a longitudinal mediation analysis. Lancet Public Health 2020; 5: e62–70. 31910981 10.1016/S2468-2667(19)30230-0

[5] Hamaker EL, Kuiper RM, Grasman RP. A critique of the cross-lagged panel model. Psychol Methods 2015; 20: 102–16. 25822208 10.1037/a0038889

[6] lecovich E Aging in place: From theory to practice. AnthropolNoteb 2014; 20: 21–32.

[7] Kall A, Jagholm S, Hesser H, Internet-based cognitive behavior therapy for loneliness: a pilot randomized controlled trial. Behav Ther 2019; published online May 11. D0I:10.1016/j.beth.2019.05.001. 32005340

[8] Bessaha ML, Sabbath EL, Morris Z, Malik S, Scheinfeld L, Saragossi J. A systematic review of loneliness interventions among non-elderly adults. Clin Soc Work J 2019; published online Oct 16. DOI:10.1007/s10615-019-00724-0.

[9] Tomasino KN, Lattie EG, Ho J, Palac HL, Kaiser SM, Mohr DC. Harnessing peer support in an online intervention for older adults with depression. Am J Geriatr Psychiatry 2017; 25: 1109–19. 28571785 10.1016/j.jagp.2017.04.015 PMC5600661

[10] Cacioppo JT, Hawkley LC, Crawford LE, Loneliness and health: potential mechanisms. Psychosom Med 2002; 64: 407–17. 12021415 10.1097/00006842-200205000-00005

